# Therapeutic Targeting of Acute Myeloid Leukemia by Gemtuzumab Ozogamicin

**DOI:** 10.3390/cancers13184566

**Published:** 2021-09-11

**Authors:** Michele Gottardi, Giorgia Simonetti, Alessandra Sperotto, Davide Nappi, Andrea Ghelli Luserna di Rorà, Antonella Padella, Marianna Norata, Maria Benedetta Giannini, Gerardo Musuraca, Francesco Lanza, Claudio Cerchione, Giovanni Martinelli

**Affiliations:** 1 Onco Hematology, Department of Oncology, Veneto Institute of Oncology IOV, IRCCS, 31033 Padua, Italy; michele.gottardi@iov.veneto.it; 2 Biosciences Laboratory, IRCCS Istituto Romagnolo per lo Studio dei Tumori (IRST) “Dino Amadori”, 47014 Meldola (FC), Italy; giorgia.simonetti@irst.emr.it (G.S.); antonella.padella@irst.emr.it (A.P.); 3 Hematology and Transplant Center Unit, Dipartimento di Area Medica (DAME), Udine University Hospital, 33100 Udine, Italy; alessandra.sperotto@asufc.sanita.fvg.it; 4 Department of Hematology and Cell Bone Marrow Transplantation (CBMT), Ospedale di Bolzano, 39100 Bolzano, Italy; daviden@hotmail.it; 5 Hematology Unit, IRCCS Istituto Romagnolo per lo Studio dei Tumori (IRST) “Dino Amadori”, 47014 Meldola (FC), Italy; marianna.norata@irst.emr.it (M.N.); mariabenedetta.giannini@irst.emr.it (M.B.G.); gerardo.musuraca@irst.emr.it (G.M.); claudio.cerchione@irst.emr.it (C.C.); 6 Hematology Unit & Romagna Transplant Network, Ravenna Hospital, 48121 Ravenna, Italy; francesco.lanza@auslromagna.it; 7 Scientific Directorate, IRCCS Istituto Romagnolo per lo Studio dei Tumori (IRST) “Dino Amadori”, 47014 Meldola (FC), Italy; giovanni.martinelli@irst.emr.it

**Keywords:** CD33, acute myeloid leukemia, gemtuzumab ozogamicin, biomarkers

## Abstract

**Simple Summary:**

Gemtuzumab Ozogamicin (GO) is a drug approved for the treatment of acute myeloid leukemia (AML). It targets leukemic cells that express the CD33 molecule on their surface and brings the toxic agent calicheamicin inside the cell to kill it. Several studies have shown that AML patients can benefit of the addition of GO to chemotherapy during induction regimens, pre- and post-transplantation. Moreover, some disease features have been addressed or are under investigation for their capacity to predict response to GO, with the future aim of selecting AML patients that can mostly benefit of GO treatment.

**Abstract:**

Acute myeloid leukemia (AML) is a complex hematological malignancy characterized by genetic and clinical heterogeneity and high mortality. Despite the recent introduction of novel pharmaceutical agents in hemato-oncology, few advancements have been made in AML for decades. In the last years, the therapeutic options have rapidly changed, with the approval of innovative compounds that provide new opportunities, together with new challenges for clinicians: among them, on 1 September, 2017 the Food and Drug Administration granted approval for Gemtuzumab Ozogamicin (GO) in combination with daunorubicin and cytarabine for the treatment of adult patients affected by newly diagnosed CD33^+^ AML. Benefits of GO-based regimens were also reported in the pre- and post-transplantation settings. Moreover, several biomarkers of GO response have been suggested, including expression of CD33 and multidrug resistance genes, cytogenetic and molecular profiles, minimal residual disease and stemness signatures. Among them, elevated CD33 expression on blast cells and non-adverse cytogenetic or molecular risk represent largely validated predictors of good response.

## 1. Introduction

Many patients affected by acute myeloid leukemia (AML) benefit of chemotherapy regimens and hematopoietic stem cell transplant (HSCT). However, progress has been modest in this therapeutic setting and huge challenges remain, mainly related to off-target cytotoxicities and to different chemoresistance mechanisms.

Immunotherapy is an innovative biological cancer therapy that exploits patient natural immune defenses to identify and eradicate cancer cells. Different types of immunotherapy have been developed, including antibody drug conjugates (ADCs) [1,2]. The advances of ADCs is to combine the specificity of a monoclonal antibody with the therapeutically benefits of chemotherapy agents [3]. Indeed, in contrast to conventional chemotherapeutics, ADCs provide superior efficacy and specificity while showing low risk of off-target cytotoxicity. Indeed, the chemotherapy particles associated with ADC remain inactive while passing through blood flow and become active only after ADC internalization. As a consequence, ADCs can increase the therapeutic window by reducing the Minimum Effective Dose (MED) along with enhancing the Maximum Tolerated Dose (MTD) [4,5,6].

Great interest in AML has been raised by the sialic acid-binding immunoglobulin-like lectin (Siglec) CD33 as a therapeutic target. Indeed, CD33 became an ideal target for the development of new ADCs due to the fact that its expression is common on the surface of AML blast cells and almost absent in normal pluripotent hematopoietic stem cells. Gemtuzumab ozogamicin (GO) is a humanized anti-CD33 monoclonal antibody covalently linked to various molecules of the cytotoxic agent N-acetyl gamma calicheamicin.

When GO binds CD33 antigen on the cell surface, the GO-CD33 complex is internalized and calichemicin molecules are released inside the cytoplasm. Active calichemicin particles intercalate DNA, thus inducing DNA damages, which, if left unrepaired, lead to cell cycle arrest and leukemic cell apoptosis. Different clinical trials have highlighted the benefit of GO on patient survival. GO is also the first antibody drug conjugate approved by the U.S. Food and Drug Administration (FDA) and the increasing knowledge of the GO metabolic pathway has improved our understanding on biomarkers of response.

In this review we summarize the clinical results obtained on CD33 targeting by GO in AML as single agent and in combination with chemotherapy, its potential benefit pre- and post-transplantation and we discuss the predictive biomarkers of therapy response.

## 2. CD33 Structure and Expression: Rationale for a Targeted Therapy in AML

CD33 is a 67 kDa glycosylated transmembrane protein belonging to the Siglec family [7]. The downstream pathway and biological functions of CD33 are still poorly understood, however Siglecs family members may regulate cytokine production, dampen inflammatory and immune responses, modulate intracellular calcium mobilization, cell adhesion, apoptosis of leukemic cells and myeloid cell maturation [8,9]. From a structural point of view, CD33 cytoplasmic tail contains two conserved immunoreceptor tyrosine-based inhibition motifs (ITIM and ITIM-like motifs) which, upon phosphorylation, promote the recruitment and activation of Src homology 2 domain-containing phosphatases 1 and 2 (SHP-1 and SHP-2). These activated SHPs further dephosphorylate various signaling molecules and suppress cellular activation [8]. The suppressor of cytokine signaling 3 (SOCS3) kinase competes with SHP-1 or SHP-2 for binding to the ITIMs. After the interaction with ITIM motifs, SOCS3 promotes the proteasome-dependent degradation of CD33, resulting in myeloid cell activation and proliferation [9].

Recently, different isoforms of CD33 gene have been identified [10]. Among them, the one missing exon 2 (CD33^∆E2^) has been predicted to have a clinical impact [11,12,13]. Briefly, this isoform lacks of the V-set domain containing the immune-dominant epitopes that represent the binding site of most CD33 antibodies, including GO.

In the healthy population, binding of antibodies recognizing the V-set domain of CD33 showed that CD33 is displayed on the surface of cells committed to the myeloid lineage, from myeloblasts to monocytes and also myelocytes and is down-regulated as the normal cells mature towards terminally-differentiated granulocytes, while it is retained on macrophages and dendritic cells [14,15]. However, in AML myeloid cells fail to differentiate, thus indicating that a high number of binding sites for CD33-specific agents are preserved and can be therapeutically exploited.

## 3. Gemtuzumab Ozogamicin: Mechanism of Cytotoxicity

Exploratory clinical studies indicated that the pharmacological inhibition of CD33 using unconjugated anti-CD33 antibodies have limited activity against AML cells.

From a structural point of view, GO is a recombinant humanized immunoglobulin G4 (IgG4) kappa (named P67.6) which specifically targets the CD33 antigen, linked to N-acetyl gamma calicheamicin, via the acid-labile hybrid 4-(4’-acetylphenoxy) butanoic acid linker. To improve the clinical applicability, researchers grafted the complementary-determining regions of P67.6 into a human immunoglubluin G4 (IgG4) kappa framework (hP67.6). Moreover, to stabilize the drug and to prevent Fab-arm exchange with endogenous human IgG4, a core-hinge mutation (S228P) was introduce in the IgG4 sequence [16,17]. Unconjugated P67.6 and hP67.6 antibodies *per se* lack substantial anti-leukemic activity, but they are useful to deliver a chemotherapy (e.g., calicheamicin derivatives) to CD33^+^ cells.

**Figure 1:**
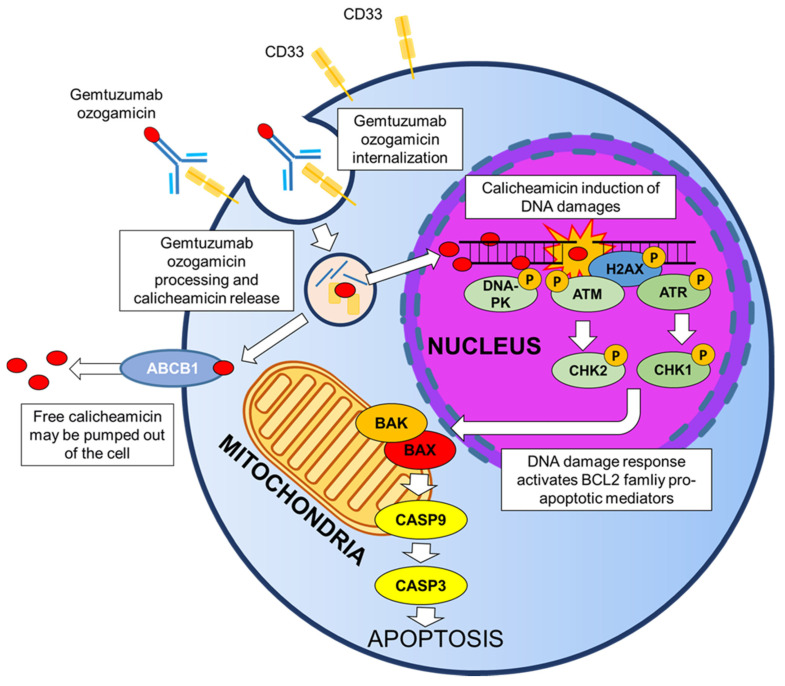
Mechanisms of action of GO.

As already mentioned, after binding to the CD33 antigen, the GO-CD33 complex is rapidly internalized (Figure 1). The 4-(4’-acetylphenoxy)butanoic acid linker that connects the antibody portion to calicheamicin is rapidly hydrolyzed in acid environment, such as the lysosomes of the myeloblast. Following the hydrolyzation, calicheamicin dimethyl hydrazide is released and reduced by the glutathione into highly reactive species, which then bind to the DNA in the minor groove. The addition of calicheamicin to the DNA structure causes site-specific damages and, in particular, double-stranded breaks which are extremely toxic for proliferating cells. Downstream, the activation of the DNA repair pathway is mediated by the ataxia-telangiectasia mutated (ATM)/ataxia-telangiectasia and Rad3-related (ATR) protein kinases [18,19] (Figure 1). In turn, ATM and ATR kinases phosphorylate CHK1 and CHK2 proteins, which induce G2/M cell cycle arrest. The DNA-dependent pathway (DNA-PK) kinase participates in the response to DNA damages by phosphorylating H2AX histones and consequently promoting the recruitment of other DNA repair mediators on the site of damage. Finally, the DNA damage is repaired throughout the homologous recombination (HR) repair or the non-homologous end joining (NHEJ) repair mechanisms. Hence, it has been showed that cancer cells defective of different DNA damage response genes (e.s. ATM or DNA-PK) are hypersensitive to calicheamicin [20,21]. If the DNA damage is not repaired, ATM/ATR kinases trigger apoptosis through the phosphorylation of two BCL2 family proteins (BAX and BAK), which release the cytochrome-c and activate caspase 9 and 3 [22,23] (Figure 1). Data from a phase II trial suggest that the inhibition of BCL2 functionality using a specific antisense (Oblimersen sodium) may enhance the induction of leukemic cell apoptosis in patients subjected to a concomitant treatment with GO [24].

Cytotoxicity analyses in the HL-60 human leukemia cell line showed a 2000-fold higher effect of the drug compared with the unconjugated calicheamicin alone [25]. In the cytoplasm, free calicheamicin can be pumped out through ATP-binding cassette (ABC) transmembrane transporters (ABCB1 and ABCC1), thus reducing its cytotoxic effect [26]. Independently from the action of transmembrane transporters, the sensitivity to calicheamicin varies significantly among AML patients [27], thus emphasizing the importance of defining the factors that regulate the anti-leukemia activity of GO. The reasons why calicheamicin sensitivity changes among patients are still unknown.

## 4. Clinical Trials and Clinical Experience of GO in AML

### 4.1. GO as Monotherapy for Newly Diagnosed or Relapsed/Refractory Adult AML

Three phase II trials (0903B1-201-US/CA [NCT00003131], 0903B1-202-EU, 0903B1-203-US/EU [NCT00003673]) assessed the safety and efficacy of GO administered as a monotherapy at 9 mg/mq on day 1 and day 14 in adult AML patients at first relapse: the results of these studies showed that GO induces a complete remission (CR) or CR with incomplete platelet recovery (CRp) in up to 25–35% of patients [26,28,29,30,31,32,33].

Based on these results on single-drug activity, on May 2000, FDA accelerated the regulatory approval of GO for CD33^+^ AML patients older than 60 years, in first relapse and unfit for intensive treatment [34].

Phase I studies identified a dose and schedule for GO (9 mg/mq every 2 weeks), able to reach complete or near complete CD33-binding site saturation while lacking dose-limiting non-hematological toxicity [26,35]. This drug schedule is however limited to the fact that the expression of new CD33 molecules is constantly displayed on blasts cell surface and thus the antigen levels, down-modulated by GO exposure, return to baseline after 72 h [35]. Fractionated GO dosing (3 mg/mq every 3 days) may therefore enhance intracellular calicheamicin delivery compared with higher dose schedules, which may be supra-saturating in some patients.

The sequential phase II/III EORTC-GIMEMA AML-19 trial first determined the best GO induction regimen. In the phase II trial GO was administered as monotherapy, 6 mg/mq on day 1 plus 3 mg/mq on day 8 versus (vs.) GO 3 mg/mq on day 1, 3 and 5, while the phase III trial compared GO to best supportive care in patients ≥61 years that were unsuitable for intensive chemotherapy [36]. A higher rate of disease non-progression, defined as CR/CRp rate or patients in stable disease at the end of induction course was reported in patients treated with the first drug schedule. Moreover, another phase III clinical trial revealed that the schedule 6 mg/mq on day 1 plus 3 mg/mq on day 8 of GO monotherapy significantly improved overall survival (OS) compared to best supportive care (1-year OS: 24.3% vs. 9.7%, hazard ratio (HR): 0.69, 95% confidence interval (CI): 0.53–0.90, *p* = 0.005) [37].

### 4.2. GO as Monotherapy for Relapsed/Refractory Pediatric AML

Regarding pediatric AML, the first phase I study of GO for compassionate use for children with relapse/refractory disease defined the effective dose of 4–9 mg/mq in up to 3 cycles in monotherapy [38]. Arceci and colleagues studied the effect of treatment with GO in monotherapy with doses of 6–9 mg/mq (2 doses, 2-week intervals) in children with relapse/refractory AML. Using this drug schedule, they found CR in 30% and 26% of AML patients with refractory and relapsed disease, respectively [39]. In 2010, a subsequent phase II study showed a significantly higher survival of children with advanced AML treated with two doses of 7.5 mg/mq of GO with 14-day intervals compared to children who did not receive the treatment (3-year probability of overall survival: 27.0% vs. 0.0%, respectively; *p* = 0.001) [40].

### 4.3. GO in Combination with Chemotherapy for Newly Diagnosed or Relapsed/Refractory Adult and Pediatric AML

During the last years, different clinical trials evaluated the efficacy of GO in combination with anti-leukemic drugs or with drug efflux pump inhibitors in both newly diagnosed or advanced AML patients. Unfortunately, due to the limited number of patients enrolled and/or absence of correct control arms, they failed to provide convincing proofs of the efficacy of GO in those settings.

Four randomized clinical trials evaluated the efficacy of GO in combination with the first cycle of intensive chemotherapy in AML patients:

SWOG S016: The Southwest Oncology Group conducted a phase III randomized trial to evaluate the clinical benefit of adding GO (6 mg/mq on day 4) to the standard 3 + 7 induction regimen in AML patients at first relapse. To balance toxicities patients allocated to the GO arm received lower dose of daunorubicin (45 mg/mq vs. 60 mg/mq) in comparison with patient treated with conventional induction regimen [41]. The interim analysis reported a higher number of fatal toxicities in the GO arm compared to the other, causing the premature end of the study and the withdrawal of GO from the market on June 2010. Moreover, the complete data analysis of the trial failed to demonstrate any clinical improvement by GO addition both in terms of relapse-free survival (RFS) and OS (GO vs. non-GO arm: 5-year RFS, 43.0% vs. 42.0%; *p* = 0.40; 5-year OS, 46.0% vs. 50.0%; *p* = 0.85).

Medical Research Council (MRC) AML15 and National Cancer Research Institute (NCRI) AML16: based on the results of a dose-finding trial that evaluated GO addition (3 mg/mq vs. 6 mg/mq) to intensive chemotherapy, with 3 mg/mq GO appearing effective and safe [42], two randomized phase III trials addressed the clinical consequences of adding GO 3 mg/mq to the induction regimen in young (predominantly ≤60 years, MRC AML15) [43] and older patients (NRCI AML16) [44]. In these trials, the GO arm showed an improved OS in elderly patients and in younger ones with favorable-risk AML.

Acute Leukemia French Association (ALFA)-0701: since the CD33 antigen is rapidly re-expressed on the surface of AML blasts after GO exposure, the acute leukemia French association tested the fractionated treatment (3 mg/mq on day 1, 4 and 7) in a phase I/II study in combination with chemotherapy for the treatment of relapsed AML [45,46]. Sixty-five to 75.0% of patients achieved CR/CRp. Based on these results, the randomized phase III ALFA-0701 trial compared fractionated GO (3 mg/mq on days 1,4 and 7 during induction and on day 1 of each consolidation course) plus standard chemotherapy vs. chemotherapy alone in newly diagnosed CD33^+^ AML patients aged 50 to 70 years [47]. The GO arm displayed an improvement of event free survival (EFS, median value 13.6 vs. −9.5 months, HR: 0.66; 95% CI: 0.49–0.89; *p* = 0.006), but no differences in terms of OS compared with the chemotherapy arm (HR: 0.81; 95% CI: 0.60–1.09; *p* = 0.16) [47,48].

Thanks to these results, on September 2017, FDA re-approved GO for adult newly diagnosed CD33^+^ AML patients and for pediatric relapsed/refractory CD33^+^ AML patients (aged ≥2 years). GO received the marketing authorization of the European Medicine Agency (EMA) on April 2018 for the treatment of newly diagnosed de novo CD33^+^ AML patients aged ≥2 years, in combination with daunorubicin and cytarabine.

A meta-analysis of 3325 AML patients treated in the above reported clinical trials showed that the addition of GO had no impact on the overall remission rate. However, it reduced the 5-year cumulative incidence of relapse with an odds ratio (OR) of 0.81 (95% CI: 0.73–0.90, *p* < 0.001) and it improved survival (OR for death = 0.90, 95% CI: 0.82–0.98, *p* = 0.01) [49].

A subsequent trial (NCRI AML17) evaluated the impact of GO dosing 3 mg/mq vs. 6 mg/mq combined with intensive chemotherapy in a cohort of 788 newly diagnosed AML patients. The result of the study showed no correlation between the increase of GO dosage and clinical benefit. Indeed, the increased GO dosing did improve neither the response rate nor the patients’ outcome (OS: HR: 1.10; 95% CI: 0.90–1.34; *p* = 0.3; RFS: HR: 1.11; 95% CI: 0.91–1.35; *p* = 0.30 [50].

### 4.4. GO-related Toxicities

Most of GO-related toxicities have been reported after first infusion. Acute infusion-related toxicities, such as chills, fever, low or high blood pressure, nausea/vomiting were the most frequently observed events. However, all these events were usually transient and could be resolved using standard interventions. The most common adverse event reported in AML patients treated with GO in monotherapy was bone marrow myelosuppression, resulting in grade 3–4 neutropenia and thrombocytopenia. In the ALFA-0701 trial, similar toxicities were seen in patients treated with GO in combination with chemotherapy. In these patients, myeloid recovery was not significantly delayed following induction with GO and chemotherapy while platelet recovery was prolonged for days [47,48]. Severe intra-vascular hemolysis have been reported in some pediatric cases treated with GO [51]. The biological reason of this phenomenon may be ascribed to impaired hemoglobin scavenging coming from the elimination of CD33^+^ monocytes/macrophages expressing the CD163 hemoglobin scavenger receptor [14]. Other transient toxicities have been reported following GO therapy and, in particular, transient alterations of liver enzymes levels including hyper-bilirubinemia and increased level of aspartate and/or alanine aminotransferase (AST/ALT) [52,53].

Among GO-related toxicities, the development of veno-occlusive disease (VOD) has the highest clinical impact. Different clinical trials highlighted that the risk of VOD in patients treated with GO is dose-dependent. Indeed, a relatively low risk of VOD has been reported in AML patients receiving doses of GO lower than 3 mg/mq and in combination with conventional therapy [50]. Accordingly, a recent study on 137 GO-treated patients and 548 matched control subjects demonstrated that GO exposure before myeloablative allogenic transplantation does not associate with higher frequency of VOD or death [54]. On the other hand, the risk of VOD increases when GO is administered in heavily pre-treated AML patients or when the doses were higher than 3 mg/mq [55,56]. The biological reason for the development of VOD are still unknown, however similar observations come from patients treated with inotuzumab-ozogamicin, an anti-CD22 calicheamicin conjugate, used for the treatment of acute lymphoblastic leukemia (ALL) patients [57]. This phenomenon suggested that the mechanism of GO-associated VOD is CD33 independent but may be related to the structure of the antibody or related to calicheamicin toxicity. More than one aspect might be involved and their effective contribution might depend on the level of CD33-binding sites saturation achieved in the blood. Defibrotide, a drug commonly used to treat VOS, provided some benefits in the treatment of GO-induced VOD, with 17/27 patients (63.0%) surviving and/or showing a response, with a safety profile comparable to the one reported in other defibrotide studies [58].

### 4.5. GO Treatment before and after Transplantation in Adult AML Patients

Risk stratification in AML is currently used to tailor patients’ post-remission treatment, which may include transplant (autologous, autoSCT or allogeneic, alloSCT) or continued chemotherapy. Post-remission treatment for AML patients should be adjusted according to an assessment of transplant-related mortality (TRM) along with leukemia characteristics and minimal residual disease (MRD).

In this context, GO could be considered before harvesting, in order to achieve and/or consolidate MRD negativity, and during transplant, for intensifying conditioning regimens: of course, a modulation of GO dosage (and of conditioning regimens) should be evaluated. A list of clinical trials including GO treatment before or after transplantation is reported in Table 1 (clinicaltrials.gov updated to 1 September 2021).

In the favorable risk core binding factor (CBF) subtype, GO was not able to reduce the residual leukemia initiating clone that survived the consolidation therapy, thus showing no benefit in the setting of autoSCT [59].

The retrospective analysis of post-transplant outcomes in subjects who received HSCT as follow-up therapy in the ALFA-0701 trial showed that fractionated-dose GO in the induction and consolidation regimen did not induce higher rate of post-transplant VOD/sinusoidal obstruction syndrome or mortality [60]. Post-transplant outcomes were comparable between arms and the study failed to demonstrate the survival benefit observed in the GO vs. control arm in patients who did not receive HSCT. Taking together, these data indicate that HSCT can follow GO-based regimen, as consolidation treatment. Accordingly, in a “real-life” setting, the combination of fractionated GO with cytarabine and mitoxantrone (MYLODAM scheme) confirmed that a GO-based intensive regimen can be applied as bridge to alloSCT in relapsed/refractory AML [61]. Moreover, the efficacy of GO combinations as a potential bridge to transplant was confirmed in a retrospective study of 24 high-risk AML patients who received fractionated GO in combination with intermediate-dose cytarabine and daunorubicin as salvage therapy [62]. A recent study also reported that relapsed AML patients may also benefit of GO monotherapy as a conditioning regimen before second alloSCT from the same donor used in the first transplantation [63]. After transplantation, the disease relapse remains the major cause of therapy failure for AML patients. Moreover, the therapeutic options to treat relapsed patients after transplant are extremely limited, due to the rising of disease resistance and a higher risk of toxicities. Therefore, there is a clinical need for therapeutic strategies able to prevent or manage disease relapse. The optimal pharmacological compound should have a safe toxicity profile, an anti-tumor effect and an immune profile, which can be used to boost GVL and reduce GVHD. Several cases of treatment with GO or GO plus donor lymphocyte infusion (DLI) for AML relapsing after alloSCT have been previously reported. The available experiences [64,65,66,67] suggest that GO treatment followed by DLI is more effective when administered soon after relapse or, if possible, even in a pre-emptive setting. A recent study reported encouraging results by fractionated GO combined with intensive chemotherapy in adult CD33^+^ AML patients relapsing after alloHCT, as salvage regimen, with an overall response rate of 72.0% and OS of 42.0% at 2 years [68]. Moreover, the combination with additional targeted therapies, when available, has to be taken into account.

### 4.6. GO Treatment before and after Transplantation in Pediatric AML

GO is currently being evaluated in this setting also in the pediatric population (Table 1). Data based on clinical experience showed that GO can be safely added (i) to a busulfan/cyclophosphamide conditioning regimen before alloSCT in children and adolescents affected by poor-risk AML [69]; (ii) to fludarabine and cytarabine (FLA) before HSCT for first-line refractory AML in children [70]; (iii) to fludarabine, cytarabine, granulocyte colony-stimulating factor and idarubicin (FLAG-IDA) as reinduction therapy before a KIR-ligand-mismatched cord blood transplant in pediatric relapsed/refractory AML [71]. Moreover, GO treatment, either as monotherapy or in combination with cytarabine or other agents, of relapsed/refractory pediatric AML patients enabled blast reduction [72] also to MRD negativity levels [73], thus allowing HSCT, without imposing major adverse events.

In the pediatric setting, GO consolidation (4.5 mg/mq to 9 mg/mq per dose) after reduced-intensity conditioning and alloSCT was safe in pediatric patients with CD33^+^ AML [74] in CR1/CR2, with OS probability at 1 and 5 years of 78.0% and 61.0%, respectively [75].

## 5. Biomarkers of Response to GO Therapy

**Figure 2:**
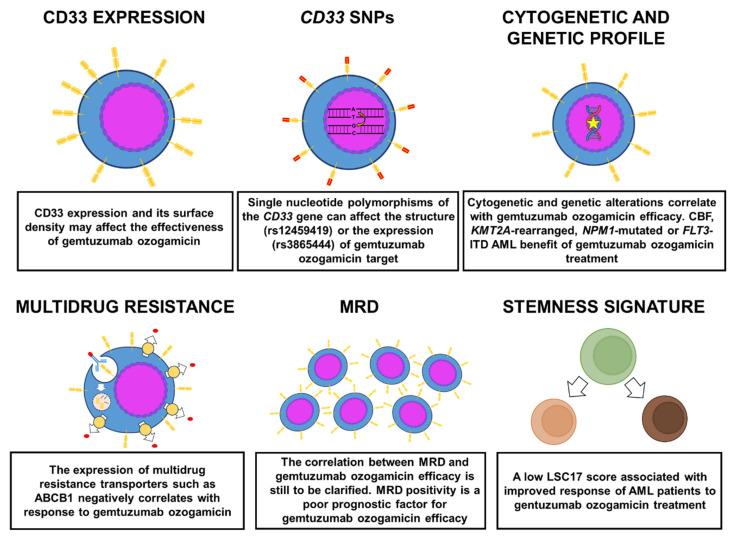
Biomarkers predictive of GO response in AML.

Since the clinical benefit of GO is variable among patients and potential adverse effects have been reported, it is important to identify factors able to predict treatment response (Figure 2).

### 5.1. CD33 Expression

The anti-leukemic effect of GO is likely correlated with the cytoplasmic level of activated calicheamicin derivates and to the intrinsic sensitivity of the target cells to DNA damage. The concentration of calichemicin molecules inside the target cells can be affected by the density of CD33 molecules on the cell surface, the efficiency of 4-(4’-acetylphenoxy) butanoic acid linker hydrolysis and the mechanism of calicheamicin activation.

CD33 is heterogeneously expressed on the surface of leukemic cells [76,77]. Data from mathematical models suggested that the cytoplasmic concentration of calichaemicin, given a fixed dose of GO, depends on the absolute number of CD33^+^ target cells, the CD33 production rate and the drug efflux pumps (ATP-binding cassette transporter) activity rather than on CD33 density on leukemic cell membrane [78]. Nevertheless, it has been showed the CD33 density on AML cells can affect GO efficacy in specific settings. In vitro studies showed that the surface CD33 expression was relevant to the drug cytotoxic effect and CD33 expression levels positively correlated with GO binding activity and leukemic cell clearance [79].

Different clinical trials investigated the relationship between CD33 density, quantified as mean fluorescence intensity or percentage of CD33^+^ blasts, and GO efficacy (Table 2):

The EORTC-GIMEMA AML-19 trial reported that GO therapy improved survival in AML patients with a percentage of CD33^+^ blasts higher than 80% [37];

In the ALFA-0701 trial, a retrospective analysis revealed that GO improved EFS and RFS of patients expressing high CD33 surface levels and the prognostic relevance was maintained when adjusting for other predictive markers such as cytogenetics and *NPM1*/*FLT3*-ITD mutational status [80];

In the MRC/NCRI trial, a significant relationship between the risk of post-GO relapse and the percentage of CD33^+^ myeloblasts has been documented. In details, patients with the lowest expression of CD33^+^ blasts were at higher risk of relapse when treated with GO compared with subjects with the highest expression, measured according to quartiles [81];

The Children’s Oncology Group (COG) AAML0531 trial reported an association between CD33 mean fluorescent intensity and response to GO. Patients belonging to the second to fourth quartiles, compared with the lowest quartile of CD33 mean fluorescence intensity, had higher CR rates, lower MRD rates at the end of the first therapy cycle, lower risk of relapse and better disease free survival (DFS) across three cytogenetic/molecular risk group [76].

Taken together, this evidence indicates that CD33 expression is an important pre-treatment biomarker of GO response and it should be used to select patients that can significantly benefit of the treatment.

### 5.2. CD33 Single Nucleotide Polymorphism

Splicing variants of the *CD33* gene generate alternative isoforms of the transmembrane receptor compromising GO binding. Molecular analysis conducted on patients from the COG AAML0531 trial indicated that *CD33* genotypes can predict response to GO therapy [82] (Table 2). Indeed, patients carrying the rs12459419 CC genotype (51.0% of patients) had a significant lower risk of relapse and better EFS and DFS when treated with GO plus chemotherapy compared with chemotherapy only. Conversely, the rs12459419 C > T (Ala14Val) polymorphism, either in heterozygosis (39.0% of cases) or homozygosis (10.0% canceled the clinical benefit of GO. The results were recently confirmed in the adult *NPM1*-mutated (mut) AML population from the AMLSG 09-09 phase III study, which compared the clinical outcome of patients receiving GO plus induction (3 mg/mq on day 1) and consolidation chemotherapy (3 mg/mq on day 1 of the first consolidation cycle) [83]. Patients with the rs12459419 CC genotype showed a superior RFS in the GO arm compared with conventional therapy. The rs12459419 C > T polymorphism resulted in *CD33* exon 2 skipping and, consequently, in the generation a shorter *CD33* isoform [84]. From a structural point of view, the above mentioned isoform lacks the immunoglobulin-like V-set domain, which is the specific antigen detected by GO and which is generally used for the quantification of CD33 level of expression in flow cytometry [82]. As a consequence, patients displaying the TT genotype, had a significantly lower CD33 expression compared to those with CT or CC genotype (TT < T < CC: *p* < 0.001) [85,86]. Similar data were reported for the rs3865444 single nucleotide polymorphism (SNP), which localizes in the non-coding, promoter region of the *CD33* gene and frequently occurs in linkage disequilibrium with rs12459419 [82,87]. In a multivariate analysis including the CD33 genotype, cytogenetic/molecular risk and CD33 surface expression, the rs12459419 CC genotype was still independently associated with lower RR and better DFS, while CD33 expression lost its significance in the evaluation of GO response [85]. This association, which deserves further investigation, could explain the differences in GO response observed among races, since the rs12459419 SNP CC genotype is more frequent in African-Americans compared with the European population [85]. Five additional CD33 SNPs identified in AML patients, namely rs1803254 (G > C; 3’UTR), rs35112940 (G > A; Arg304Gly), rs2455069 (A > G; Arg69Gly), rs61736475 (T > C; Ser305Pro) and rs201074739 (CCGG deletion), can impact on GO response [88]. A reduced RR was observed in GO treated patients carrying rs1803254 GG (*p* = 0.009), rs35112940 GG (*p* < 0.001), rs2455069 GG (*p* = 0.005), rs1736475 TT (*p* = 0.002) and rs201074739 CCGG/CCGG (*p* = 0.002).

Interestingly, a recent composite score, namely the CD33_PGx6_Score, based on six CD33 SNPs (rs12459419, rs2455069, rs201074739, rs35112940, rs61736475 and rs1803254), has been established to understand the impact of CD33 SNPs on CD33 expression and on GO efficacy [88]. The analysis, conducted on 938 patients from the COG AAML0531 cohort, showed that a CD33_PGx6_Score higher than 0 associated with high CD33 expression, better RFS (5-year RFS: 62.5% vs. 46.8% in the GO arm compared to the control arm; *p* = 0.008) and lower RR (5-year RR: 28.3% vs. 49.9% in the GO arm compared to the non-GO arm; *p* < 0.001). Conversely, the addition of GO did not affected on the outcome of AML patients having a CD33_PGx6_Score below 0.

### 5.3. Cytogenetic Alterations

Data from randomized clinical trials evaluating the effect of GO and intensive chemotherapy in adults AML patients highlighted that the efficacy is correlated with the cytogenetic risk (Table 2). In details, the addition of GO was associated with a survival benefit of 20.7% and 5.7% in patients from good (OR: 0.47; 95% CI: 0.31–0.73, *p* < 0.001) and intermediate cytogenetic risks (OR: 0.84; 95% CI: 0.75–0.95, *p* = 0.005) groups, respectively [49]. Conversely, GO did not improve clinical outcome in the adverse cytogenetic group, that generally express lower levels of CD33, with an absolute survival benefit at 6 years of 2.2% (OR: 0.99; 95% CI: 0.83–1.18, *p* = 0.90). Several studies corroborated the clinical benefit of GO addition in non-adverse cytogenetic risk patients [37,43,47,89].

CBF and *lysine methyltransferase 2A* (*KMT2A)*-rearranged AML, that belong to the good and intermediate/high risk classes, respectively, deserve further discussion.

CBF-AMLs are characterized by low CD33 expression [90], which can be predictive of poor GO response. The low CD33 expression is likely related to the cellular stage of differentiation. Indeed, t(8;21)/inv(16)/t(16;16) arise in preleukemic CD33^−^ cells at a very early stage of differentiation [91] and this cells may be spared by GO treatment. Conversely, additional alterations inducing a proliferative status occur late in CD33 expressing cells, that can be effectively targeted by GO. However, a phase II trial studying the efficacy in adult CBF-AML patients of the FLAG induction regimen as frontline therapy in combination with GO 3 mg/mq at induction day 1 and post-remission course 1 and 2 day 1 (FLAG-GO), showed that FLAG-GO induced a higher remission rate (95.0%) and it was associated with a 3-year OS and RFS of 78.0% and 85.0%, respectively [92]. In particular, the addition of GO to standard chemotherapy was demonstrated to mitigate the risk of relapse in CBF cases carrying mutations in the exon17 of the *KIT* gene [93]. The benefit of GO addition was also confirmed by comparing FLAG-GO with FLAG-Idarubicin, in terms of molecular response rate (76.0% vs. 42.0%, *p* = 0.002) and 5-year RFS (87.0% vs. 68.0%, *p* = 0.02) [94].

11q23/*KMT2A*-rearrangements, which characterize 4% of adult [95] and 15–20% of pediatric AML [96], associate with high CD33 expression in leukemic cells [97]. Despite being classified as adverse risk leukemia, in most cases *KMT2A*-rearranged AML had a good response to GO in relapsed/refractory patients [98,99]. The analysis of 215 *KMT2A*-rearranged AML from the COG AAML0531 trial revealed that patients treated with GO in combination with chemotherapy achieved higher EFS compared with those receiving chemotherapy alone (5-year EFS: 48.0% vs. 28.0%, *p* = 0.002) [100].

These differences may be due to diverse blast sensitivity to calicheamicin (e.g., high sensitivity in CBF cells) and, potentially, drug uptake into AML cells [92,97]. Indeed, ABC transporter activity is generally high in elderly AML patients and in adults with adverse-risk cytogenetics [101,102]. This may explain the poor response to GO therapy of adult patients characterized by adverse-risk cytogenetics. In this scenario, the poor response rate may be also relate to the lower cell surface density of CD33 in these cases. Notably, the cytogenetic/molecular risk did not affect OS and DFS in a phase II multicenter clinical trial that enrolled 130 patients, aged < 65, treated with FLAI-GO induction regimen. The study showed a CR rate of 82.0% after induction [103]. The probability of 1, 2, and 5-year DFS was 77.0%, 58.0% and 52.0%, respectively, with a median follow-up of 54 months. Age and molecular remission after FLAI-GO and alloSCT predicted prolonged DFS in a Cox multivariate analysis. These data raises new hopes on the combination of GO with induction and consolidation chemotherapy regimens (other than cytarabine and doxorubicin only) for the treatment of high-risk patients.

### 5.4. Molecular Profile

The molecular profile is commonly used to stratify AML patients into prognostic subgroups when receiving standard chemotherapy [95]. Similar approaches can be exploited also when using newly approved antileukemic drugs.

Recently, Fournier and colleagues evaluated the predictive value of molecular alterations on the efficacy of combining GO with standard frontline chemotherapy [104]. By analyzing data from the ALFA-0701 trial, they confirmed that only patients classified into favorable (HR 0.54; 95% CI: 0.30–0.98) and intermediate (HR 0.57; 95% CI: 0.33–1.00) risk categories according to the European LeukemiaNet (ELN) 2017 risk stratification could benefit of GO combined with conventional chemotherapy. Conversely, the outcome of patients belonging to the adverse risk group was not affected by GO (HR 0.93; 95% CI: 0.61–1.43), in line with data obtained by cytogenetic risk classification [49,91] (Table 2).

When focusing on individual gene mutations, data from the literature indicate that *NPM1*-mut (25–35% of AML patients in general and 45–60% of cytogenetically normal AML) or *FLT3*-ITD patients (~20% of cases) had a higher CD33 expression compared to wildtype (wt) cases [80,105,106] and may benefit from GO treatment. In the ALFA-0701 trial, a clinical benefit was observed on 2-year EFS, RFS and OS in the *NPM1*-mut cohort [47]. The AMLSG 09-09 study did not confirm the result in terms of 2-year EFS (HR: 0.83, 95% CI: 0.65–1.04; *p* = 0.1), however GO treatment reduced the incidence of relapse in patients achieving CR/CR with incomplete hematologic recovery (HR: 0.66; 95% CI: 0.49–0.88; *p* = 0.005) [107]. In this cohort, GO also improved 2-year EFS of *FLT3* wild-type, but not *FLT3*-ITD mut patients (HR: 0.72; 95% CI: 0.56–0.95 vs. HR: 1.53; 95% CI: 0.95–2.48, respectively; *p* = 0.002). Similar results regarding *FLT3* mutational status and response to GO were obtained in adult AML patients from the MRC AML15 and NCRI AML16 trials, in which GO failed to demonstrate a clinical improvement in *FLT3*-ITD cases [49]. In this cohort, GO benefit on *NPM1*-mut cases was not seen either. This outcome may be partially attributed to the administration of a single GO dose in the MRC AML15 and NCRI AML16 trials. In contrast, other studies reported an improved OS, EFS and RFS in adult AML patients with *FLT3*-ITD by the addition of GO [47,89,104]. The analysis of *FLT3*-ITD patients from the COG AAML03P1 and AAML0531 studies revealed a decreased RR in those treated with GO-based regimens (37.0% vs. 59.0%, *p* = 0.02) [108]. In particular, the incidence of relapse was significantly reduced in patients that were exposed to GO prior of undergoing a HSCT in first CR (22.0% vs. 56.0%, *p* = 0.003). Interestingly, the cohort of poor prognosis patients harboring a high *FLT3*-ITD allelic ratio, that was exposed to GO prior to transplant, had a lower RR (15.0% vs. 53.0%, *p* = 0.007).

In the ALFA-0701 trial the benefit of GO was not limited to *FLT3*-ITD patients, but it was extended to all cases with activating signaling mutations (*FLT3*-ITD, *FLT3*-TKD, *KRAS*, *NRAS*, S, *PTPN11*, *JAK2*, *RIT1*, *CBL*, HR 0.43; 95% CI: 0.28–0.65), which correlated with higher CD33 expression levels [104]. Notably, mutant *PTPN11* was shown to confer resistance to the BCL-2 inhibitor venetoclax via upregulating MCL1, pMCL1, and BCL-xL [109], thus indicating that GO-based regimens may be a valuable therapeutic strategy for this subgroup of patients. Elevated CD33 expression was also measured in patients carrying co-occurrent mutations in epigenetic modifiers and signaling genes (98.0% vs. 60.0% of CD33^+^ cells in patients carrying both mutation types and altered epigenetic regulators only, respectively; *p* < 0.001). No association was reported between CD33 expression and mutations of *NPM1* or spliceosome genes in this study.

The link between activating signaling mutation and GO benefit is not clear. This observation is substantiated by the good response of CBF patients [92,94], that frequently harbors mutations in signaling genes [110], to GO treatment, despite the low CD33 expression [90]. As already mentioned, CD33 is a transmembrane glycoprotein receptor whose downstream pathway results in an inhibitory effect on myeloid cells [8]. Therefore, the co-occurrence with mutations in signaling genes may be an oncogenic mechanism adopted by leukemic cells to overcome CD33 downstream effects [111].

In contrast to the above results, the GOELAMS/FILO AML 2006-IR trial, enrolling younger AML patients (<60 years) with intermediate cytogenetic risk, failed to achieve a clinical improvement by the addition of GO to chemotherapy, even by stratifying cases according to their molecular profile [112]. Unsupervised analysis of patients according to the mutational status of seven genes (*NPM1*, *FLT3*-ITD, *CEBPA*, *DNMT3A*, *IDH1*, *IDH2*, *ASXL1*) identified six mutational clusters defining three major outcome groups (group A: *NPM1*-mut, *FLT3*-ITD-wt or biallelic *CEBPA*-mut; group B: no mutations, or *NPM1*-mut *FLT3*-ITD or *NPM1*-mut, *FLT3*-ITD-wt, epigenetic mutations; group C: *NPM1*-wt, *FLT3*-ITD). Although the results may not be conclusive, due to GO-related toxicity that led to early closure, group C showed the worst OS, DFS and EFS, followed by group B, while group A displayed the best outcome, when considering either patients receiving standard chemotherapy or those treated by chemotherapy plus GO.

Overall, these data suggest that the benefit of GO can be dependent on the disease genetic background, with patients carrying mutations in signaling genes and high CD33 expression being candidate for a better response. The analysis of the mutational status of a large panel of AML-related genes may help define genetic backgrounds predicting a clinical benefit related to GO addition to chemotherapy regimens and may guide novel tailored treatment strategies. For example, current trials are evaluating the relevance of the use of GO in combination with targeted therapies [33], including FLT3 inhibitors (NCT03900949, NCT04385290, NCT04293562) and PARP inhibitor (NCT04207190).

In addition to the genetic profile, the transcript levels of the anticoagulant factor ANXA5 were shown to predict better OS and EFS in multivariate analysis in pediatric AML receiving GO in combination with conventional chemotherapy [113]. By dividing the cohort according to the median ANXA5 expression, patients showing high ANXA5 levels had significantly better OS (*p* = 0.0012) and EFS (*p* < 0.001) compared with the other group.

### 5.5. Multidrug Resistance

The function of the ABCB1 multidrug resistance protein has been reported as a mechanisms dampening in vitro GO-mediated apoptosis [114,115,116]. Despite its pro-apoptotic effects, it is not surprising that free calicheamicin may be a substrate of the ABCC1 transporter [115,117,118]. Although similar results were obtained when GO is administered in combination with intensive chemotherapy, formal proofs are still lacking. 58% of AML patients expresses ABCB1 and its frequency in blasts cells varies from 19.0% to 75.0% of positive cells [118,119,120]. Several studies reported an association between ABCB1 and poor in vivo GO response, including failure to clear bone marrow blasts and to achieve CR [118,121,122,123], or dismal outcome, as indicated by OS and EFS [115,119,124,125] (Table 2). This data was confirmed in a cohort of pediatric relapsed/refractory AML patients, in which the relative degree of ex vivo drug efflux was predictive of GO response in the clinical setting. Indeed, 5 out of 8 patients with low drug efflux ratios achieved CR/CRp, that was not in patients with high drug efflux levels [39]. Other studies suggested that GO response may be also shaped by the inter-individual variability of calicheamicin efflux level [27]. Moreover, the *ABCB1* genotype was shown to have a clinical impact on GO response, by acting on the accumulation of calicheamicin. A recent analysis on 942 patients from the COG AAML0531 cohort reported that GO recipients with rs1045642 (C > T; Ile1145Ile) CT or TT genotype displayed a better outcomes compared with those displaying the CC genotype (CT or TT vs. CC, 5-year EFS: *p* = 0.022; 5–year RR: *p* = 0.007), which correlated with an increased intracellular calicheamicin retention [123].

### 5.6. Minimal Residual Disease

Monitoring MRD after induction even in patients achieving morphological CR has prognostic impact in AML patients [126,127,128]. Therefore, different molecular or immunophenotypic markers have been studied to monitor MRD also in response to GO treatment. In the NCRI AML16 trial, the MRD level was measured by flow cytometry in order to evaluate its role as independent prognostic factor of response. No significant correlation has been identify between MRD negativity and the administration of GO in comparison to the control arm (57.0% vs. 48.0%; *p* = 0.18) [128].

*NPM1* mutation and Wilms’ tumor 1 (*WT1*) gene expression are suitable prognostic molecular markers for MRD analysis by quantitative reverse-transcription polymerase chain reaction [129]. The ALFA-0701 study showed that negativity for *NPM1* mutation MRD was frequently achieved in GO-treated patients compared to the control arm, both at the end of induction (39.0% vs. 7.0%; *p* = 0.006) and end of treatment (91.0% vs. 61.0%; *p* = 0.028) [129]. Conversely, GO provided no advantage when MRD was measured by *WT1* transcript level at both time points (MRD negativity, after induction: 75.0% vs. 65.0%; *p* = 0.29; at the end of treatment 82.0% vs. 80.0%; *p* = 1.0).

Recently, the prognostic impact of *NPM1* mutation MRD in AML patients receiving GO was determined in the phase III AMLSG 09-09 trial, that analyzed 7526 samples, including peripheral blood and bone marrow, from 469 patients [130]. At the end of cycle 2 and at the end of treatment, a reduction ≥1000 of *NPM1* mutation transcript combined with MRD negativity was predictive of lower incidence of relapse. Patients receiving GO plus chemotherapy achieved lower levels of transcript level of *NPM1* mutation across all treatment cycles compared with those treated with standard chemotherapy, thus resulting in a higher frequency of MRD negative cases at the end of treatment (56.0% vs. 41.0%; *p* = 0.01). Moreover, MRD positive patients belonging to the GO arm showed lower levels of *NPM1* mutation transcript after two treatment cycles, which in turn led to lower RR (29.3% vs. 45.7% at 4-years; *p* = 0.009).

Overall, these data indicate that MRD is a valuable tool to measure GO response and that a combination strategy based on GO and chemotherapy can increase the rate of patients achieving MRD negativity (Table 2).

### 5.7. Stemness Signature

Leukemic stem cells (LSC) are the main determinants of AML refractoriness and relapse. The main features responsible for therapy resistance include cell cycle quiescence, self-renewal and high levels of drug efflux [131]. Recently, a 17-gene expression signature for LSC has been established in AML, named the LSC17 score, that has prognostic value [132]. In particular, its application to the ALFA-0701 trial data revealed that GO addition improved the outcome of patients having low LSC17 score (EFS: HR: 0.42; *p* = 0.001; RFS: HR: 0.53; *p* = 0.03), but not those with high signature score. These results indicate that the LSC17 score can serve as biomarker to predict response of AML patients to GO (Table 2).

**Table 2:** Main evidence of in vivo GO efficacy in AML subgroups defined according to biomarkers of response.

Biomarker	Main Observations	Study	References
CD33 expression	GO improved OS of patients with >80% CD33^+^ blasts	EORTC-GIMEMA AML-19	[37]
	GO improved EFS and RFS of patients expressing high CD33 surface levels	ALFA-0701	[80]
	Low percentage of CD33^+^ blasts associated with higher risk of relapse after GO	MRC AML15, NCRI AML16	[81]
	Patients in the 2nd to 4th quartiles of CD33 surface expression had higher CR and lower MRD rates at the end of the first cycle, lower risk of relapse and better DFS	COG AAML0531	[76]
CD33 SNPs	rs12459419 CC genotype: lower risk of relapse and better EFS and DFS in the GO arm	COG AAML0531	[82]
	rs12459419 C > T SNP: no GO benefit	COG AAML0531	[82]
	*NPM1*-mut patients with the rs12459419 CC genotype showed a superior RFS in the GO arm	AMLSG 09-09	[83]
	Patients carrying rs1803254 GG, rs35112940 GG, rs2455069 GG, rs1736475 TT and rs201074739 CCGG/CCGG had reduced RR to GO	COG AAML0531	[88]
	CD33_PGx6_Score >0 associated with high CD33 expression, better RFS and lower RR in the GO arm	COG AAML0531	[88]
Cytogenetic alterations	GO provided a survival benefit in patients with good and intermediate cytogenetic risks, but not in the adverse cytogenetic group	Various	[37,43,47,52,89,91]
	The addition of GO to standard chemotherapy reduced the risk of relapse in CBF cases carrying *KIT* mutations	FLAG-GO	[93]
	The addition of GO to standard chemotherapy induced higher EFS in *KMT2A*-rearranged AML	COG AAML0531	[100]
Molecular profile	GO provided a survival benefit in patients from favorable and intermediate, but not adverse molecular risk categories (ELN 2017)	ALFA-0701	[49,91]
	GO provided EFS, RFS and OS benefit in *NPM1*-mut AML and reduced the incidence of relapse in *NPM1*-mut patients achieving CR/CRi	ALFA-0701, AMLSG 09-09	[50,107]
	GO improved EFS of *FLT3*-ITD-wt, but not *FLT3*-ITD-mut patients	AMLSG 09-09, MRC AML15, NCRI AML16	[52,107]
	GO improved OS, EFS and RFS and reduced the RR in adult *FLT3*-ITD-mut patients	COG AAML03P1, COG AAML0531	[50,92,107,108]
	GO provided clinical benefit to patients with activating signaling mutations	ALFA-0701	[104]
	The mutational status of seven genes identified a group characterized by *NPM1*-mut, *FLT3*-ITD-wt or biallelic *CEBPA*-mut that displayed the best outcome in the GO arm	GOELAMS/FILO AML 2006-IR	[112]
Multidrug resistance	ABCB1 expression associated with failure to clear bone marrow blasts and to achieve CR or poos OS and EFS	Various	[115,118,119,121,122,123,124,125]
	*ABCB1* rs1045642 CT or TT genotype associated with better outcomes in GO recipients	COG AAML0531	[123]
MRD	GO-treated patients frequently achieved negativity for *NPM1* mutation MRD and a reduction 1000 of *NPM1* mutation transcript combined with MRD negativity was predictive of lower RR	ALFA-0701, AMLSG 09-09	[130,131]
LSC signature	GO addition improved the outcome of patients having low LSC17 score but not those with high signature score	ALFA-0701	[132]

CR: complete remission; CRi: complete remission with incomplete hematologic recovery; DFS: disease free survival; EFS: event free survival; LSC: leukemia stem cell; MRD: minimal residual disease; mut: mutated; OS: overall survival; RFS; relapse free survival; RR: relapse rate; SNP: single nucleotide polymorphism.

## 6. Novel Preclinical GO-based Therapeutic Combinations in AML

**Figure 3:**
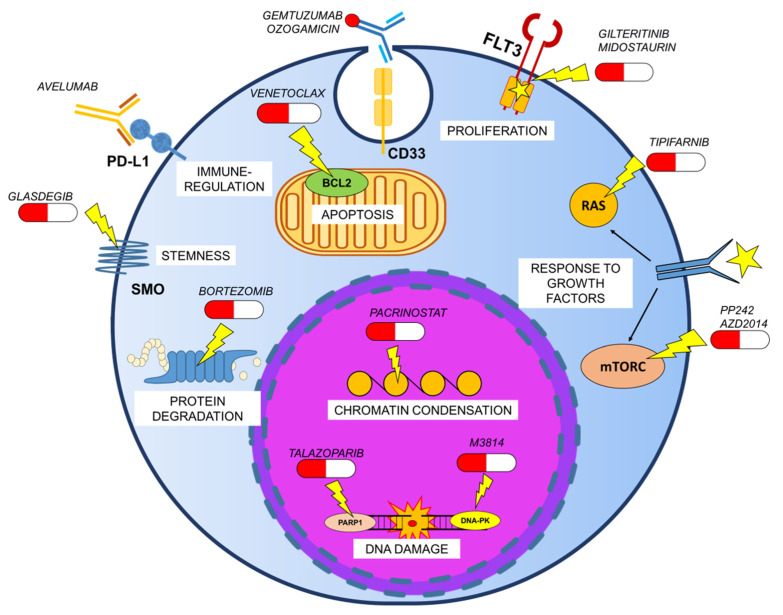
Targeted agents that are under clinical or preclinical investigation in combination with GO.

In addition to strategies based on standard chemotherapy regimens, GO is under clinical investigation in association with novel drugs (e.g., CPX-351 liposome), the demethylating agent azacytidine and/or targeted therapies, including venetoclax, avelumab, FLT3 inhibitors (midostaurin, gilteritinib), glasdegib, bortezomib, pacrinostat, talazoparib, that are variably combined between each other and with nucleoside analogs and/or anthracyclines (Figure 3) [33].

Moreover, insights for novel and future therapeutic combinations come from preclinical studies. Non-cytotoxic concentrations of the PP242 [133] or the combination with AZD2014 [134], two mTORC1/2 dual kinase inhibitors, enhanced GO cytotoxicity in AML cells, by potentiating lysosomal functions and suppressing GO-induced CHK1 activation, thus promoting cell cycle progression with damaged DNA and, ultimately, cell death. DNA damage accumulation is also at the base of the successful combination of GO with the farnesyltransferase inhibitor tipifarnib/zarnestra. It has been showed that farnesyltransferase inhibitors induce DNA damage generating reactive oxygen species [135]. In particular, the CD34^+^CD38^−^ phenotype and cell dormancy, that impaired DNA damage resolution, were predictive of higher chemosensitivity to the drug combination ex vivo [136].

Great interest is currently raising around the combination of GO with DNA repair inhibitors. Since calicheamicin induces DNA double strand breaks, agents that block the non-homologous end joining (NHEJ) pathway are expected to synergize with GO treatment. This hypothesis has been confirmed by combining GO with the inhibitor of DNA-PK (M3814) [137] or with the PARP inhibitors olaparib [138] and talazoparib [139].

## 7. Conclusions

CD33 represents a *bona fide* target in AML therapy, since it is highly expressed in the majority of leukemic cells. GO, targeting CD33, is not a perfect drug, due its conjugation technology and susceptibility to depotentiation by ABC transporter activity. The clinical history of GO in oncology has been long and unusual. After an accelerated approval for AML patients, GO was withdrawn by the company in 2010 following the confirmatory phase III clinical trial, in which GO did not show a clear clinical benefit in comparison to control arms. Indeed, the study was stopped prematurely after the high number of early deaths occurred in the arm receiving GO in comparison with those receiving chemotherapy alone. However, in light of the widespread CD33 expression on AML cells and its specificity for the myeloid lineage, GO was an interesting drug in the field. GO came back into the market seven year later after the promising results of the spontaneous ALFA-0701 trial, in which novel GO drug schedule and concentration demonstrated superiority in favorable and intermediate risk patients. A better knowledge of the disease biology and its molecular subtypes was crucial for the definition of a target population that could benefit of GO-based regimens.

Over the past years, the advancements of sequencing technologies, strategies for MRD monitoring, genotyping studies and the increased knowledge of the mechanisms of therapy resistance have expanded the number and type of biomarkers able to predict GO response. Future studies on large cohorts should try to combine the diverse biomarkers to improve the prediction accuracy, thus paving the way for precision medicine in the real-life clinical practice.

Moreover, the development of novel targeted agents paves the way to new combinatory therapies that may enhance GO efficacy. Indeed, ongoing studies are investigating the role of GO in in patients’ subpopulations and in newer therapy combinations, towards more personalized and, hopefully, less toxic, approaches. Currently, studies open for enrollment are mainly focused on GO treatment in elderly patients, in combination with demethylating agents and other targeted drugs. In this clinical setting, drug benefits, that display a high level of variability among patients, according to known biomarkers and target-specific toxicity affecting normal tissue, which may be a limit to GO administration, have to be taken into account carefully. The regulatory history of GO is a hallmark of the potential pitfalls that may occur in the drug development process towards clinical application.

## Figures and Tables

**Table 1:** Clinical trials of GO used before or after transplantation (clinicaltrials.gov updated to 1 September 2021).

NCT Number	Intervention	Conditions	Age	Phase	Trial Status
NCT00044733	GO at relapse after auto or alloSCT	AML	child, adult, older adult	II	Completed
NCT02221310	GO+chemotherapy followed by alloSCT	high-risk AML/MDS	up to 25 years	II	Recruiting
NCT00669890	GO+Busulfan and Cyclophosphamid before alloSCT	high-risk AML/MDS/JMML	up to 30 years	I	Terminated
NCT02117297	GO consolidation after alloSCT	average-risk AML/MDS	up to 25 years	II	Recruiting
NCT01020539	GO consolidation after alloSCT	average-risk AML/MDS/JMML	up to 30 years	I	Active, not recruiting
NCT00460447	GO before alloSCT at relapse	AML	18–70 years	I/II	Unknown
NCT00038831	GO+Melphalan+Fludarabine before alloSCT in older or medically infirm patients	AML/MDS/CLL	12–75 years	I/II	Completed
NCT00476541	GO consolidation after SCT	AML	up to 18 years	III	Completed
NCT00008151	GO+fludarabine+total-body irradiation before alloSCT	advanced AML/MDS	child, adult, older adult	II	Completed
NCT00038805	GO+nonmyeloablative preparative regimen before mini-alloSCT in older or medically infirm patients	AML/ALL/CML/MDS	55–75 years	II/III	Terminated
NCT01723657	GO “in vivo purging” before autoSCT in patients with favorable/intermediate characteristics and without matched related donor	AML	18–70 years	II	Completed
NCT00070174	GO in remission induction, intensification therapy before alloSCT	AML	child, adult, older adult	II	Completed

Allo: allogenic; AML: acute myeloid leukemia; auto: autologous; CLL: chronic lymphocytic leukemia; CML: chronic myeloid leukemia; GO: gemtuzumab ozogamicin, JMML: juvenile myelomonocytic leukemia; MDS: myelodysplastic syndrome.

## Data Availability

Not applicable.
